# Calculated globulin as a surrogate marker for hypogammaglobulinemia: establishing clinical decision limits in a Brazilian population cohort

**DOI:** 10.3389/fimmu.2026.1743499

**Published:** 2026-05-08

**Authors:** André L. O. Feodrippe, Gabriela Negritu, Ana C. Guersoni, Cristina M. Kokron

**Affiliations:** 1 Division of Clinical Immunology and Allergy, Faculdade de Medicina, Universidade de São Paulo, São Paulo, Brazil; 2 CSL Behring Bern, Bern, Switzerland; 3 CSL Behring Brazil, Sao Paulo, Brazil; 4 Hospital Israelita Albert Einstein, São Paulo, Brazil

**Keywords:** antibody deficiencies, calculated globulin, clinical decision limits, clinical outcomes, hypogammaglobulinemia, primary immunodeficiencies

## Abstract

**Introduction:**

Inborn errors of immunity (formerly primary immunodeficiencies) encompass a group of genetic disorders characterized by defects in innate and/or adaptive immunity. Serum Immunoglobulin G (IgG) quantification is the diagnostic gold standard for hypogammaglobulinemia, but it is not routinely ordered in general clinical practice. Calculated globulin (CG) has been proposed as a potential surrogate marker. The current study aimed to evaluate the association between CG levels and adverse clinical outcomes and to propose CG-based clinical decision thresholds relevant to a cohort of Brazilian population.

**Methods:**

A cross-sectional analysis of longitudinal records was conducted encompassing CG measurements from 50, 539 individuals aged ≥1 year. Odds ratios (OR) and probability curves were calculated using predefined CG cut-off points (0.5 to 2.1 g/dL).

**Results:**

Linear regression analysis demonstrated a linear association between CG and IgG levels, described by the equation IgG = 0.523 × CG − 0.33. Lower CG levels were associated with increased risk of mortality, hospitalization, ICU admission, emergency care utilization, and higher frequency of antibiotic use. A CG threshold of ≤2.0– 2.1 g/dL was associated with a higher risk of adverse outcomes.

**Discussion:**

CG is a widely available, low-cost parameter with potential utility as a screening tool for primary or secundary antibody deficiencies. Clinical decision thresholds for hypogammaglobulinemia appear to be higher than the lower reference limits currently used in Brazilian laboratories. These findings highlight the need for revised clinical decision limits tailored to specific populations and support the integration of CG into routine screening strategies for earlier detection of antibody deficiencies.

## 1 Introduction

Inborn errors of immunity (IEI), formerly known as primary immunodeficiencies, encompass nearly 550 distinct disorders, most of which are hereditary and monogenic (1, 2). Their defining characteristic is a dysfunction of innate and/or adaptive immune responses. Clinically, they present increased susceptibility to infections, autoimmunity, autoinflammation, allergies, and malignancies (2). Previously considered solely as conditions that increased susceptibility to infectious diseases, they are now also known to commonly present with non-infectious manifestations marked by immune dysregulation. These features contribute to a broad spectrum of clinical phenotypes, making the diagnostic process challenging. Advancements in clinical knowledge and the broadening of the genetic landscape of these diseases have facilitated a more refined characterization of their non-infectious manifestations and established stronger genotype–phenotype correlations (3, 4).

Although individually rare, IEIs are collectively more common than previously recognized. Considering ethnic variations, their prevalence in the general population is conservatively estimated at between 1 in 1, 000 and 1 in 5, 000 individuals. These conditions involve heterogeneous immunologic abnormalities and present a highly variable clinical spectrum (5). Particularly in populations with low levels of consanguinity, the most predominant deficiencies are those of antibodies, with more than 50% of overall cases. From the age of two, predominantly antibody deficiencies represent the most observed subgroup of inborn errors of immunity (6). The spectrum ranges from severe dysfunction, with a reduction in all immunoglobulins and the absence of B cells, to specific antibody deficiencies, with normal levels of all immunoglobulins classes and IgG subclasses (7). However, disturbances in antibody production/function also occur in other subgroups of IEI, making this immunological disorder even more widespread. There are only a few epidemiologic data on the Brazilian population, and, if the prevalence patterns reported in international studies persist, we may estimate that between 40, 000 and 200, 000 individuals have an immune system defect, with 20, 000 to 100, 000 of these cases involving antibody deficiencies (7).

In most cases, there is a significant delay in diagnosis. While the majority of primary immunodeficiencies manifest during childhood, over 50% of cases are diagnosed in patients older than 25 years (8). Delayed recognition and treatment of IEI result in increased morbidity, a decline in quality of life characterized by frequent emergency room visits and hospitalizations, and higher mortality rates. Among other publications, a study in a Brazilian cohort showed that in patients with primary hypogammaglobulinemia, the frequency and severity of infections decreased after a specific therapeutic intervention, reducing lung complications (9). The frequent exacerbations, as well as treatment of anatomical sequelae such as bronchiectasis often observed in these patients, determine higher costs for the healthcare system (10, 11).

The lack of awareness among physicians, even in major national centers and among specialists, remains a significant barrier to improved diagnosis and patient care (12, 13).

Clinical strategies to reduce diagnostic delays include continuous medical education and the broad dissemination of warning signs suggestive of immunodeficiencies. The “10 warning signs” of primary immunodeficiency, identified through statistical analysis, are also applicable to secondary immunodeficiencies (14, 15). Major barriers to early diagnosis include the wide spectrum of clinical manifestations and the difficulty in effectively communicating this information across various specialists and levels of healthcare (16).

Population-based immunologic screening approaches in the laboratory have already proven highly successful, for example, in newborn screening for severe combined immunodeficiency (SCID), looking for T-cell receptor excision circles (TRECs) in DNA extracted from Guthrie spots, and in agammaglobulinemia, looking for kappa deletion recombination B-cell excision circles (KRECs) (17). However, the establishment of opportunistic laboratory screening strategies to detect other antibody deficiencies, whether secondary or primary, including Common Variable Immunodeficiency (CVID), which often presents later in life, continues to represent a significant challenge (8, 18).

Serum IgG level measurement remains the most specific method for confirming and diagnosing hypogammaglobulinemia, however, it is rarely requested by non-specialist physicians. Decreased IgG levels can interfere with various laboratory tests, and the determination of the calculated globulin (CG) is a promising parameter for a screening strategy.

Calculated globulin (CG) is determined by subtracting serum albumin from total protein, two analytes commonly measured as part of the clinical workup for nutritional status and hepatic function (19, 20). Given that protein and albumin measurements are routinely performed, often even in primary care, the derivation of CG is widely feasible. Within this framework, diverse clinical indications - including liver function assessment, nutritional evaluation, and paraproteinemia screening - collectively yield vast repositories of laboratory data amenable to secondary analysis. This accumulation of information can be framed within the concept of ‘big data, ’ a term that - although defined with some variation across the literature - is generally applied to describe large and continuously generated datasets distinguished by their considerable volume, rapid velocity, and limited structural organization (21).

Initially observed in the adult population of Wales, a Brazilian study confirmed the existence of a linear relationship between IgG and CG levels, which was also found in pediatric populations of different age groups (19, 22). Analyzing the distribution of CG levels in a large cohort will be useful in defining reference ranges for a subset of the population, which may eventually be extrapolated to the broader Brazilian population.

The interpretation of laboratory parameters from any specimen inherently relies on comparison - either with prior results from the same individual or with established normative values. Reference intervals are designed to define the expected range of results in healthy populations, bounded by lower and upper limits (23). It is essential, however, to differentiate reference intervals from clinical decision limits, the latter being anchored in associations with specific disease outcomes and risks and determined by the anticipated benefit of timely intervention (24).

As previously noted, the clinical manifestations of IEI are highly variable, with increased susceptibility to infections being a major hallmark. These infections are often severe, show poor response to standard antibiotic therapy, and frequently require hospitalization and escalation to intravenous antibiotics (25).

In this context, the present study aims to evaluate clinical outcomes - such as mortality, hospital admission, Intensive Care Unit (ICU) admission, and antibiotic use - in individuals aged 1 year and older who underwent total protein and protein fraction testing. The worse clinical outcomes may suggest that those patients might have a dysfunctional antibody production, whatever the cause. The aim is to propose potential age-stratified clinical decision limits for calculated globulin (CG), thereby suggesting alert thresholds to prompt confirmatory measurement using the gold standard method which is serum immunoglobulin quantification.

## 2 Methods

### 2.1 Study design

The study was designed as a cross-sectional analysis of longitudinal laboratory and clinical records collected between January 2018 and December 2023 from routine clinical testing across all clinical laboratory units of Hospital Israelita Albert Einstein, a private hospital in São Paulo, Brazil. The first available measurement of calculated globulin for each individual was defined as the baseline exposure to ensure independence of observations. Clinical outcomes documented in the electronic medical record were longitudinally ascertained throughout the study period, including mortality, hospitalization, ICU admission, and urgent and/or emergency admissions, antibiotic usage, immunoglobulin administration, and the average number of visits.

### 2.2 Study population and sample selection

The study population consisted of a convenience sample derived from routine clinical laboratory testing, representing a dynamic population with variable observation time across individuals.Individuals under 1 year and with documented human immunodeficiency virus (HIV) infection were excluded. To minimize potential bias related to disorders affecting protein metabolism (protein-losing conditions, chronic inflammatory diseases, or hepatic and renal dysfunction), subjects with abnormal serum albumin values were also excluded. Due to distinct reference intervals of albumin levels for each age group, the samples were categorized into subgroups of 1–7 years, 8–14 years, 15–17 years, and those over 18 years.

### 2.3 Laboratory analytical methods

The analytes were collected from the serum of patients and processed within the stability time. Blood was collected from the patient into a drytube, centrifuged and immediately processed and analyzed using Cobas 6000 platform. The samples were processed using colorimetric methods, bromocresol green for albumin and biuret for total protein made by Roche. This method was standardized by linear regression of 2 points against reference IRMM BCR470 CRM470 for albumin and linear calibration of 2 points against SRM927 for total protein. Serum immunoglobulin concentrations were determined in a subset of patients according to their physician´s clinical indication, using the Roche immunoturbidimetric immunoglobulin assay, following the manufacturer’s instructions and standardized laboratory procedures (26).

### 2.4 Statistical analysis

Cut-off points for calculated globulin (CG) were defined based on thresholds previously reported in national and international studies, using values of 0.5 g/dL, 1.0 g/dL, 1.5 g/dL, 1.8 g/dL (27), 1.9 g/dL (28), 2.0 g/dL (29), and 2.1 g/dL to stratify individuals into comparison groups. For each threshold, individuals with CG values below the defined cut-off were compared with those above it. Risk analyses for clinical outcomes, including mortality, hospitalization, intensive care unit admission, and urgent or emergency visits, were performed using odds ratios (OR). Associations with immunoglobulin replacement therapy were evaluated using Fisher’s exact test, while differences in the average number of healthcare visits and prescribed antibiotics were assessed using the Mann–Whitney test. Event probabilities were estimated according to the proportion of outcomes relative to the sample size and adjusted for the Charlson Comorbidity Index. In addition, linear regression analysis was conducted to evaluate the association between the first calculated globulin measurement and the first immunoglobulin G (IgG) levels among individuals for whom both measurements were available.

### 2.5 Ethical aspects

The study was approved by the ethics committee of Hospital Israelita Albert Einstein (CAAE: 82870224.4.0000.0071) and followed the principles described in the Declaration of Helsinki.

## 3 Results

### 3.1 Demographic data

Initially, 111, 530 patients who underwent total protein and fractions tests and/or protein electrophoresis at the clinical laboratory of Hospital Israelita Albert Einstein (HIAE) were identified. A total of 1, 970, 000 tests of this type were carried out during the study period. After applying the exclusion criteria, 95, 160 patients comprised the final study cohort, of whom 50, 539 had calculated globulin measurements available for analysis, most of them derived from outpatient settings (n=27, 535).

**Figure 1:**
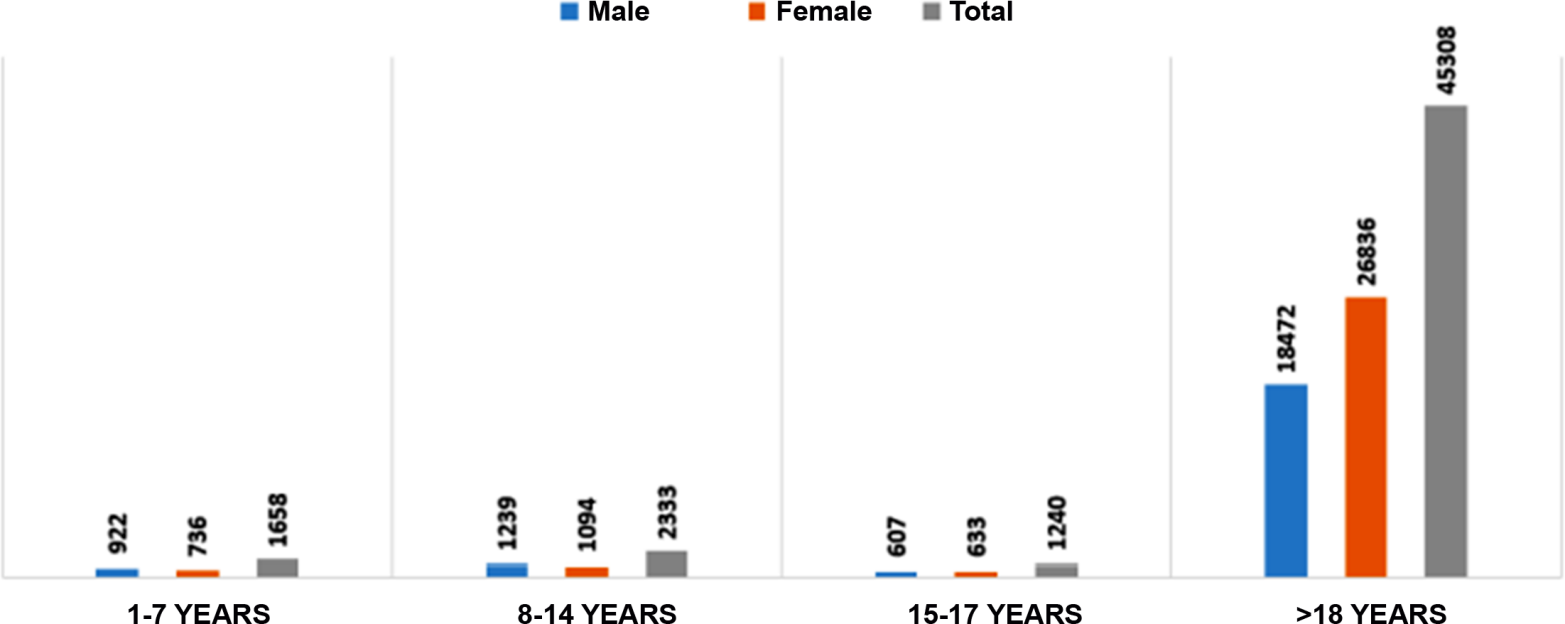
Distribution of the number of individuals with globulin tests calculated stratified by age group and sex.

Analyses were stratified by age group: 1–7 years (n = 1, 658; 922 males, 736 females), 8–14 years (n = 2, 333; 1, 239 males, 1, 094 females), 15–17 years (n = 1, 240; 607 males, 633 females), and >18 years (n = 45, 308; 18, 472 males, 26, 836 females), as illustrated in **Figure 1**.

### 3.2 Relationship between CG and IgG

**Figure 2:**
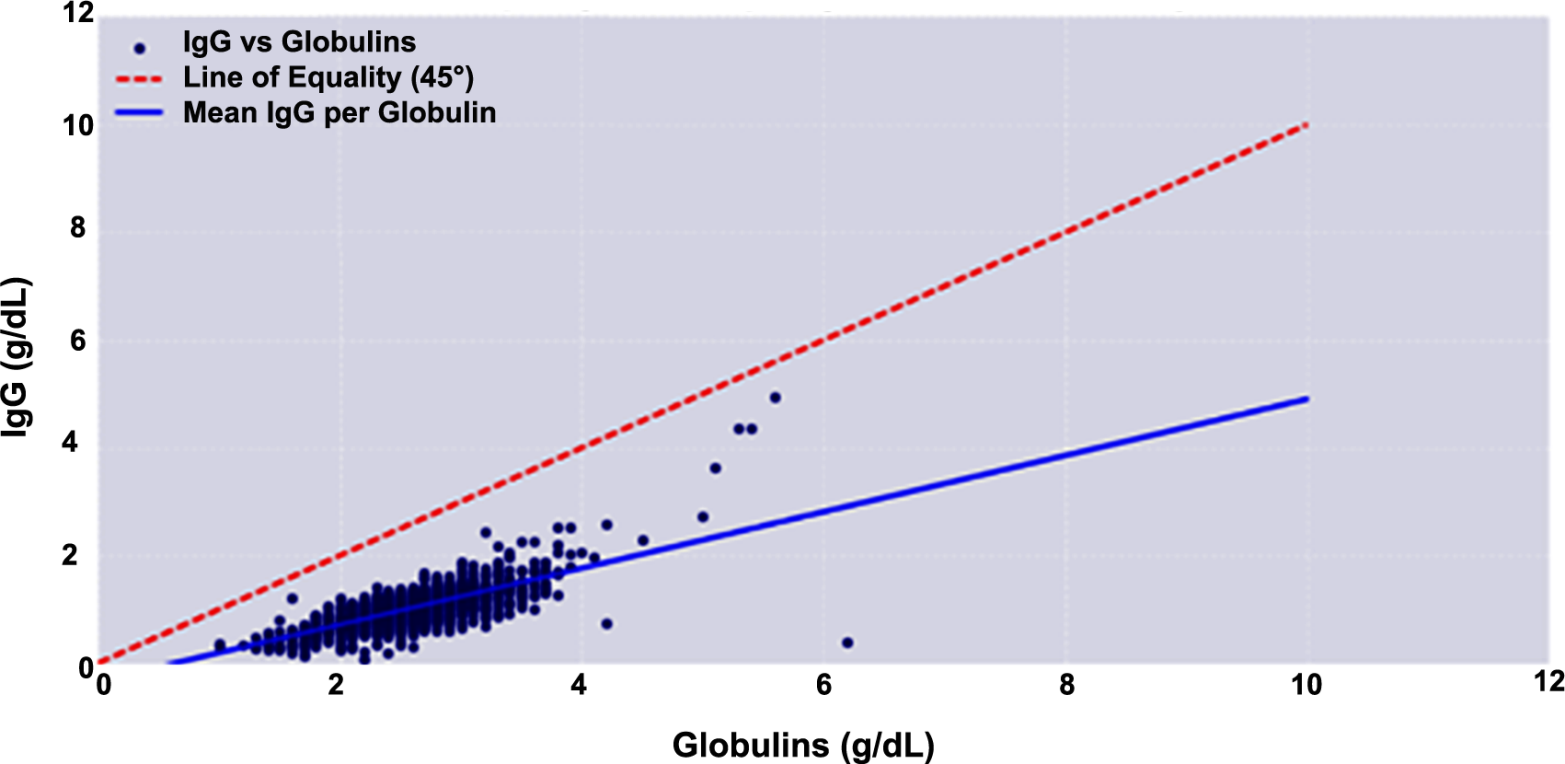
Linear regression analysis between immunoglobulin G (IgG) levels and the first available calculated globulin (CG) measurement.

The initial analysis employed linear regression to compare the first IgG concentrations(n= 7281 individuals) with the first available CG measurement for each patient, irrespective of sex or age, provided that both test results were available. The resulting equation, “IgG = 0.523 × Globulin − 0.33, ” describes the relationship between these two parameters (**Figure 2**).

### 3.3 Correlation of CG with clinical outcomes

**Figure 3:**
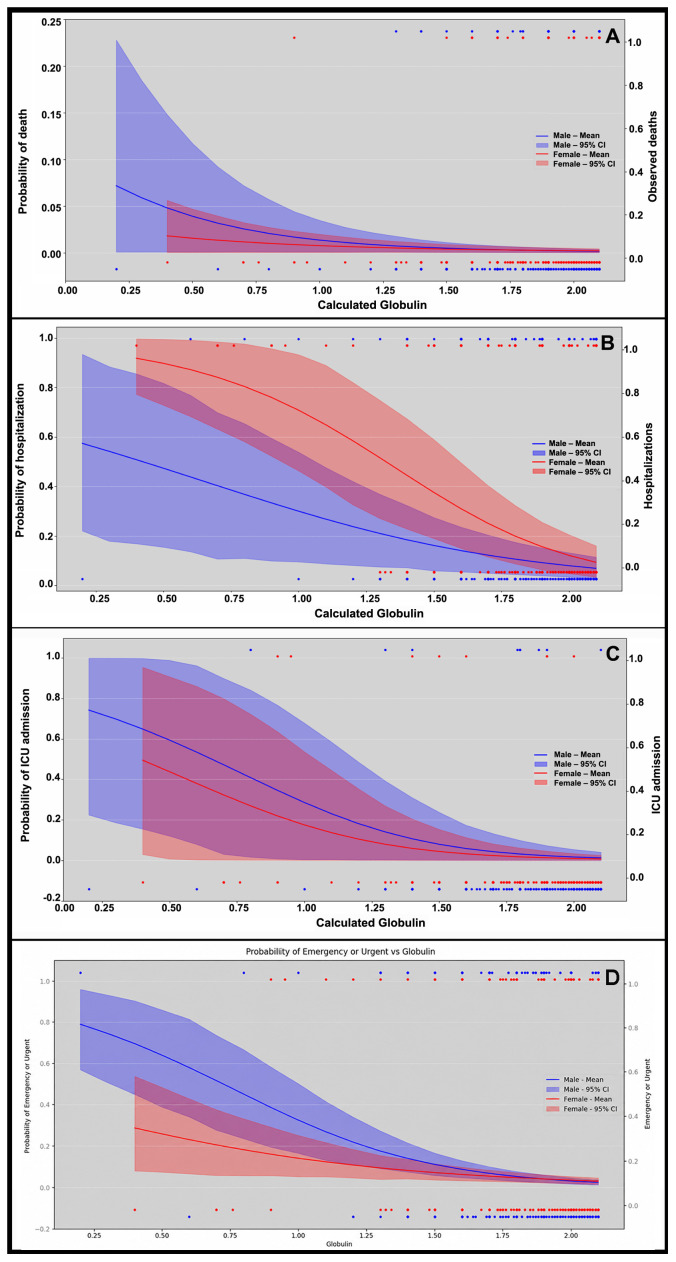
Clinical outcomes according to calculated globulin (CG) levels. **(A)** Probability of death increased with lower CG. **(B)** Probability of Hospitalization: risk rose with declining CG. **(C)** Probability of ICU admission: more likely at lower CG. **(D)** Urgent/emergency sample collection showed increased risk with lower CG.

As shown in **Figure 3A**, the probability of death increased progressively as CG levels declined. The odds ratio (OR) analysis stratified by age and sex demonstrated that, among patients aged ≥18 years, lower CG values were significantly associated with a higher risk of death (95% CI). In contrast, no significant OR differences were observed in the 8–14 and 15–17-year age groups, due to the absence of death events in these ranges (**Supplementary Table 1**). Among women ≥18 years, the risk of death was consistently elevated for all globulin levels below 2.1 g/dL. In men, a significantly higher risk of death was identified when calculated globulin levels fell below 2.0 g/dL compared with higher values.

Regarding hospitalization, patients with lower CG levels showed a higher likelihood of admission compared with those presenting higher values (**Figure 3B**). The odds ratio (OR) analysis confirmed a significant association between reduced CG and increased hospitalization risk. Owing to the larger number of events for this outcome, it was possible to detect age- and sex-specific patterns. In the 8–14 and 15–17-year groups—particularly among females—the probability of hospitalization rose progressively as globulin levels declined (**Supplementary Table 2**). Among girls aged 1–7 years, a significantly higher OR for hospitalization was observed at CG levels below 1.9 g/dL, whereas in boys of the same age group, even values up to 2.1 g/dL were associated with increased risk. In adults, hospitalization risk was elevated at CG levels up to 2.1 g/dL, thresholds still considered within the currently accepted reference range.

Lower CG levels were also associated with a higher likelihood of ICU admission (**Figure 3C**). In both the 1–7 and >18-year groups, decreasing CG values correlated with elevated odds ratios (ORs). Among adults, levels below 2.1 g/dL were linked to an increased risk of ICU admission in both sexes, whereas in girls aged 1–7 years, the risk became evident only at levels below 2.0 g/dL. No ICU admissions were reported in the 15–17-year age group (**Supplementary Table 3**).

Another variable used to estimate patient prognosis was the context of laboratory test collection—elective versus urgent or emergency settings. It was hypothesized that samples obtained under urgent or emergency conditions would reflect greater clinical severity. The probability curve revealed an increased likelihood of low globulin levels being associated with urgent/emergency testing (**Figure 3D**). In the OR analysis, patients >18 years with lower CG levels showed a higher risk, whereas no significant differences were observed among those aged 1–7 years. In the 8–14 and 15–17-year groups, significant associations were detected only at very low globulin thresholds: <1.8 g/dL in females and <1.9 g/dL (8–14 years) or <2.0 g/dL (15–17 years) in males (**Supplementary Table 4**).

Given that antibody deficiencies are commonly associated with infections, mostly by encapsulated extracellular bacteria, the occurrence of pneumonia (ICD-10 J15), acute sinusitis (ICD-10 J01), meningitis (ICD-10 G03) and acute diarrhea (ICD-10 A09), was evaluated under the hypothesis that lower calculated globulin (CG) levels would be associated with a higher prevalence of these conditions [20]. **Supplementary Table 5** presents the prevalence of infectious disease codes (ICDs) stratified by age, sex, and CG levels. No cases were identified in the 8–14 and 15–17-year groups, precluding prevalence estimates in these categories. To reduce potential biases related to underreporting of ICD codes, the mean number of antibiotic prescriptions per patient was also analyzed (**Supplementary Table 6**). Statistically significant differences were observed in specific subgroups: boys aged 1–7 years with CG <1.8 g/dL, women >18 years with CG <2.0 g/dL, and men >18 years with CG <1.9 g/dL.

It was hypothesized that patients undergoing a greater number of laboratory tests would be more likely to have active pathological conditions, thus reflecting their overall health or disease status. Based on this assumption, individuals with lower calculated globulin (CG) levels were expected to show a higher frequency of visits to laboratory collection units. To evaluate this, the mean number of visits per patient was analyzed by age, sex, and CG levels. However, this hypothesis was not confirmed, as no significant differences were observed in the mean number of visits or sample collections (**Supplementary Table 7**). In groups with no representatives, the mean number of visits was zero.

The final parameter evaluated, used as an indirect indicator of underlying immune dysfunction, was the therapeutic use of human immunoglobulin. The analysis was stratified by age, sex, and calculated globulin (CG) levels (**Supplementary Table 8**). In all age groups and both sexes, there was a consistent trend toward greater use of human immunoglobulin at lower CG levels, supporting its association with a higher prevalence of antibody-related disorders.

**Table 1:** Suggested lower thresholds of calculated globulin (CG) for adverse clinical outcomes

Female	Death	Hospitalization	ICU	Antibiotic use	ICD	Urgency/Emergency Admission	IVIgRT use	Mean of visits
1-7 years	<1.0 g/dL	<1.9 g/dL	<2.0 g/dL	<1.5 g/dL	<1.5 g/dL	NA	<1.5 g/dL	NA
8-14 years	NA	<2.0 g/dL	<1.8 g/dL	<1.9 g/dL	NA	<1.8 g/dL	NA	NA
15-17 years	NA	<2.1 g/dL	NA	<1.9 g/dL	<2.0 g/dL	<1.8 g/dL	<1.9 g/dL	NA
>18 years	<2.1 g/dL	<2.1 g/dL	<2.1 g/dL	<1.9 g/dL	NA	<2.1 g/dL	<1.5 g/dL	<1.8 g/dL
								

Male	Death	Hospitalization	ICU	Antibiotic use	ICD	Urgency/Emergency **Admission**	IVIgRT use	Mean of visits
1-7 years	<1.0 g/dL	<2.1 g/dL	<2.1 g/dL	<1.5 g/dL	<2.0 g/dL	NA	NA	NA
8-14 years	NA	<1.9 g/dL	NA	<1.8 g/dL	NA	<1.9 g/dL	NA	NA
15-17 years	NA	NA	NA	NA	NA	<2.0 g/dL	NA	NA
>18 years	<2.0 g/dL	<2.1 g/dL	<2.1 g/dL	<1.9 g/dL	<2.1 g/dL	<2.1 g/dL	<1.9 g/dL	<1.8 g/dL

ICU: Intensive Care Unit; ICD: International Classification of Diseases; IVIgRT: Intravenous Immunoglobulin replacement therapy.

### 3.4 Outcome-derived clinical decision thresholds for CG

Based on the analysis of all clinical outcomes, specific lower thresholds of calculated globulin (CG) were identified as potential early warning markers to aid clinical decision-making. **Table 1** summarizes the authors’ proposed thresholds for each outcome derived from the collected data. Particularly in adults, both sexes, values approaching 2.1 g/dL emerged as a consistent threshold associated with most adverse outcomes.

## 4 Discussion

The WHO report *“Principles and Practice of Screening for Disease”* (Wilson and Jungner, 1968) established core criteria for effective screening, including early detection, availability of beneficial intervention, and the use of accessible and reliable diagnostic methods (30). Calculated globulin (CG), derived from routinely performed and low-cost laboratory tests, fulfills several of these requirements and represents a pragmatic candidate for opportunistic screening (19, 27, 31).

Previous studies have consistently demonstrated an association between CG and serum immunoglobulin G (IgG), the reference standard for diagnosing hypogammaglobulinemia (27–29). Early investigations showed that approximately half of samples with CG <1.8 g/dL were associated with IgG <4 g/L (19, 32, 33), and that a CG value of 1.8 g/dL corresponded to an IgG concentration of approximately 6 g/L using the bromocresol green method. Our findings are consistent with this relationship, supporting the biological plausibility of CG as a marker of humoral immune status.

However, most previous studies have focused on analytical correlations or selected populations enriched for immunodeficiency, limiting generalizability. In contrast, our study evaluates CG in a large, unselected population and relates its levels to clinically meaningful outcomes. Lower CG levels were consistently associated with increased risks of mortality, hospitalization, and ICU admission, even after adjustment for comorbidities. These associations were particularly evident in adults, the largest subgroup analyzed. Notably, increased risk was observed at CG levels within or close to currently accepted reference intervals.

These findings highlight a fundamental distinction between reference intervals and clinical decision thresholds. Reference intervals describe the distribution of values in a presumed healthy population, whereas decision thresholds should reflect levels associated with adverse outcomes. Our results suggest that, for CG, these constructs are not aligned, and that clinically relevant risk may be present within ranges currently considered normal. In this context, CG may function as a risk marker rather than solely a descriptive laboratory parameter.

Evidence from Brazil has largely been derived from populations recruited in Allergy and Immunology centers, where the prevalence of immunodeficiency is higher and immunoglobulin replacement therapy may introduce confounding (23). In such settings, a CG threshold of 2.4 g/dL identified IgG <6 g/L with high diagnostic performance (34). Notably, this value exceeds currently adopted reference limits (35) and is concordant with the outcome-based thresholds observed in our study, supporting the need to reassess existing reference intervals.

From a clinical perspective, earlier identification of antibody deficiencies is associated with reduced morbidity and improved survival (10, 11, 36). CG should therefore not be considered a substitute for immunoglobulin measurement, but rather an accessible screening marker that can prompt further investigation. Reduced CG levels may serve as an initial alert, guiding targeted immunoglobulin testing and facilitating earlier diagnosis of underlying immune dysfunction.

Given that total protein and albumin measurements are already embedded in routine clinical practice, CG offers a scalable and low-cost opportunity for opportunistic screening without additional laboratory burden. This may be particularly relevant in settings with limited access to specialized immunological testing.

The robustness of our findings is supported by the exclusion of major confounders, including HIV infection and nutritional disorders, and by the proximity of our values to the lower limit of reference intervals established by the first gold-standard direct study for this analyte (37).Nevertheless, as an observational analysis, these results reflect associations rather than causal relationships, and the impact of CG-based screening on clinical outcomes remains to be determined.

Future studies should evaluate CG performance in well-characterized cohorts of primary and secondary immunodeficiencies and assess its role within structured screening strategies. Such efforts will be essential to validate and refine clinically actionable thresholds and to determine the extent to which CG-guided approaches can improve patient outcomes.

### 4.1 Limitations

This study was based on a retrospective analysis of a convenience sample derived from routine clinical laboratory testing, which may introduce selection bias. The study population represents a dynamic cohort, predominantly composed of individuals undergoing laboratory testing in an outpatient setting, with variable observation times, and standardized follow-up was not available for all individuals, preventing time-to-event analyses. To minimize bias related to comorbidities, probability adjustments were performed using the Charlson Comorbidity Index, although odds ratio (OR) analyses were also calculated without this correction. Outcomes were limited to events recorded in the electronic medical record of a single institution, and events occurring in other healthcare facilities may not have been captured. Immunoglobulin measurements were available only for a subset of individuals, which may introduce verification bias. Finally, part of the study period overlapped with the COVID-19 pandemic, and as a mitigation strategy, individuals with this diagnosis were flagged to account for potential confounding effects.

## 5 Conclusion

Calculated globulin may represent a practical and widely accessible screening marker for hypogammaglobulinemia. Our findings suggest that clinically relevant decision thresholds are higher than the lower reference limits currently adopted by Brazilian laboratories, supporting the need to reassess these limits and consider CG as an opportunistic screening parameter to prompt earlier immunoglobulin testing.

## Data Availability

The original contributions presented in the study are included in the article/**Supplementary Material**. Further inquiries can be directed to the corresponding author.
