# Supplementary material for: Calculated globulin as a surrogate marker for hypogammaglobulinemia: establishing clinical decision limits in a Brazilian population cohort
Source: Front Immunol. 2026 May 8;17:1743499. doi: 10.3389/fimmu.2026.1743499 (PMC13193802; doi:10.3389/fimmu.2026.1743499)
Supplement: Supplementary file 1 [file Table1.docx]

**Supplementary Table 1.** Odds ratios (ORs) for death according to calculated globulin (CG) levels, stratified by age group and sex. No deaths occurred in the 8–14 and 15–17-year groups, precluding OR estimates.

| **Female** | - 1. **years** | **8-14 years** | **15-17 years** | **> 18 years** |
| --- | --- | --- | --- | --- |
| <0.5 g/dL | NA | NA | NA | NA |
| <1.0 g/dL | 72.8 – [5.66, 936.56] | NA | NA | 9.29 – [1.16, 74.51] |
| <1.5 g/dL | 8.23 – [0.73, 92.36] | NA | NA | 3.63 – [0.88, 15.08] |
| <1.8 g/dL | 2.58 – [0.23, 28.61] | NA | NA | 5.01 – [3.15, 7.97] |
| <1.9 g/dL | 1.73 – [0.16, 19.2] | NA | NA | 4.04 – [2.77, 5.89] |
| <2.0 g/dL | 1.2 – [0.11, 13.23] | NA | NA | 2.81 – [2.02, 3.91] |
| <2.1 g/dL | NA | NA | NA | 2.17 – [1.62, 2.9] |
| >2.1 g/dL | NA | NA | NA | NA |
|  |  |  |  |  |
| **Male** | **1-7 years** | **8-14 years** | **15-17 years** | **> 18 years** |
| <0.5 g/dL | NA | NA | NA | NA |
| <1.0 g/dL | 64.57 – [8.5, 490.55] | NA | NA | NA |
| <1.5 g/dL | NA | NA | NA | 7.19 – [2.79, 18.55] |
| <1.8 g/dL | NA | NA | NA | 2.32 – [1.41, 3.81] |
| <1.9 g/dL | NA | NA | NA | 2.13 – [1.45, 3.13] |
| <2.0 g/dL | NA | NA | NA | 1.77 – [1.28, 2.44] |
| <2.1 g/dL | NA | NA | NA | 1.23 – [0.91, 1.64] |
| >2.1 g/dL | NA | NA | NA | NA |

Confidence Interval = 95%; NA: Not Applicable
