# Supplementary material for: Calculated globulin as a surrogate marker for hypogammaglobulinemia: establishing clinical decision limits in a Brazilian population cohort
Source: Front Immunol. 2026 May 8;17:1743499. doi: 10.3389/fimmu.2026.1743499 (PMC13193802; doi:10.3389/fimmu.2026.1743499)
Supplement: Supplementary file 2 [file Table2.docx]

**Supplementary Table 2.** Odds ratios (ORs) for hospitalization according to calculated globulin (CG) levels, stratified by age group and sex. Increased risk was observed at lower CG levels, with significant associations in both pediatric and adult groups.

| **Female** | - 1. **Years** | **8-14 years** | **15-17 years** | **> 18 years** |
| --- | --- | --- | --- | --- |
| <0.5 g/dL | NA | NA | NA | NA |
| <1.0 g/dL | 18.28 – [3.3, 101.19] | NA | NA | NA |
| <1.5 g/dL | 4.91 – [2.49, 9.7] | NA | NA | 26.72 – [14.65, 48.73] |
| <1.8 g/dL | 2.34 – [1.38, 3.96] | 1.5 – [0.2, 11.46] | 11.17 – [2.83, 44.08] | 5.58 – [4.18, 7.46] |
| <1.9 g/dL | 1.95 – [1.19, 3.2] | 3.98 – [1.48, 10.74] | 11.23 – [3.34, 37.74] | 4.26 – [3.37, 5.38] |
| <2.0 g/dL | 1.55 – [0.96, 2.49] | 2.9 – [1.23, 6.82] | 7.06 – [2.41, 20.63] | 3.07 – [2.52, 3.74] |
| <2.1 g/dL | 1.3 – [0.82, 2.06] | 2.01 – [0.93, 4.36] | 3.71 – [1.31, 10.54] | 2.34 – [1.97, 2.78] |
| >2.1 g/dL | NA | NA | NA | NA |
|  |  |  |  |  |
| **Male** | **1-7 years** | **8-14 years** | **15-17 years** | **> 18 years** |
| <0.5 g/dL | NA | NA | NA | NA |
| <1.0 g/dL | 18.56 – [6.34, 54.32] | NA | NA | 25.82 – [4.31, 154.67] |
| <1.5 g/dL | 6.56 – [3.83, 11.26] | 48.53 – [7.89, 298.64] | NA | 6.41 – [3.1, 13.28] |
| <1.8 g/dL | 2.99 – [1.93, 4.63] | 9.21 – [3.76, 22.57] | NA | 3.08 – [2.29, 4.15] |
| <1.9 g/dL | 2.31 – [1.53, 3.5] | 3.96 – [1.69, 9.27] | NA | 2.53 – [2.0, 3.21] |
| <2.0 g/dL | 2.45 – [1.65, 3.63] | 2.08 – [0.9, 4.79] | 0.65 – [0.09, 5.0] | 2.14 – [1.76, 2.61] |
| <2.1 g/dL | 1.98 – [1.35, 2.92] | 1.09 – [0.5, 2.39] | 1.14 – [0.33, 3.98] | 1.83 – [1.56, 2.16] |
| >2.1 g/dL | NA | NA | NA | NA |

Confidence Interval = 95%; NA: Not Applicable
