# Supplementary material for: Calculated globulin as a surrogate marker for hypogammaglobulinemia: establishing clinical decision limits in a Brazilian population cohort
Source: Front Immunol. 2026 May 8;17:1743499. doi: 10.3389/fimmu.2026.1743499 (PMC13193802; doi:10.3389/fimmu.2026.1743499)
Supplement: Supplementary file 3 [file Table3.docx]

**Supplementary Table 3.** Odds ratios (ORs) for ICU admission according to calculated globulin (CG) levels, stratified by age group and sex. Increased risk was observed in adults with CG <2.1 g/dL and in young girls with CG <2.0 g/dL; no ICU admissions occurred in the 15–17-year group.

| **Female** | - 1. **Years** | **8-14 years** | **15-17 years** | **> 18 years** |
| --- | --- | --- | --- | --- |
| <0.5 g/dL | NA | NA | NA | NA |
| <1.0 g/dL | 18.05 – [1.89, 172.1] | NA | NA | 589.32 – [111.74, 3108.16] |
| <1.5 g/dL | 14.11 – [3.66, 54.42] | NA | NA | 249.72 – [76.23, 818.04] |
| <1.8 g/dL | 6.65 – [1.77, 25.03] | 16.97 – [1.7, 169.67] | NA | 40.76 – [13.86, 119.91] |
| <1.9 g/dL | 7.14 – [1.78, 28.75] | 7.24 – [0.74, 70.75] | NA | 36.2 – [13.09, 100.12] |
| <2.0 g/dL | 4.89 – [1.22, 19.63] | 3.57 – [0.37, 34.64] | NA | 24.36 – [8.82, 67.27] |
| <2.1 g/dL | 3.17 – [0.79, 12.72] | 5.49 – [0.77, 39.15] | NA | 13.48 – [4.88, 37.21] |
| >2.1 g/dL | NA | NA | NA | NA |
|  |  |  |  |  |
| **Male** | **1-7 Years** | **8-14 years** | **15-17 years** | **> 18 years** |
| <0.5 g/dL | 28.22 – [2.44, 326.72] | NA | NA | NA |
| <1.0 g/dL | 14.7 – [3.78, 57.21] | NA | NA | 230.59 – [24.68, 2154.53] |
| <1.5 g/dL | 10.41 – [3.84, 28.25] | NA | NA | 90.28 – [25.41, 320.69] |
| <1.8 g/dL | 7.87 – [2.96, 20.92] | 7.27 – [0.83, 63.91] | NA | 15.98 – [5.82, 43.84] |
| <1.9 g/dL | 5.34 – [2.02, 14.14] | 3.48 – [0.4, 30.17] | NA | 16.41 – [6.78, 39.71] |
| <2.0 g/dL | 6.18 – [2.17, 17.63] | 1.91 – [0.22, 16.44] | NA | 8.86 – [3.67, 21.42] |
| <2.1 g/dL | 4.97 – [1.62, 15.25] | 0.87 – [0.1, 7.49] | NA | 5.96 – [2.51, 14.15] |
| >2.1 g/dL | NA | NA | NA | NA |

Confidence Interval = 95%; NA: Not Applicable
