# Supplementary material for: Calculated globulin as a surrogate marker for hypogammaglobulinemia: establishing clinical decision limits in a Brazilian population cohort
Source: Front Immunol. 2026 May 8;17:1743499. doi: 10.3389/fimmu.2026.1743499 (PMC13193802; doi:10.3389/fimmu.2026.1743499)
Supplement: Supplementary file 4 [file Table4.docx]

**Supplementary Table 4.** Odds ratios (ORs) for urgent or emergency laboratory test collection by CG levels, stratified by age group and sex. Significant associations were detected mainly in adults and at very low CG thresholds (<1.8–2.0 g/dL) in pediatric groups.

| **Female** | - 1. **Years** | **8-14 years** | **15-17 years** | **> 18 years** |
| --- | --- | --- | --- | --- |
| <0.5 g/dL | NA | NA | NA | NA |
| <1.0 g/dL | 0.44 – [0.05, 3.8] | NA | NA | 4.34 – [0.9, 20.91] |
| <1.5 g/dL | 1.66 – [0.9, 3.06] | NA | NA | 5.24 – [2.64, 10.42] |
| <1.8 g/dL | 1.3 – [0.88, 1.92] | 3.17 – [1.15, 8.76] | 4.34 – [1.16, 16.3] | 5.41 – [4.2, 6.96] |
| <1.9 g/dL | 1.11 – [0.79, 1.57] | 1.79 – [0.79, 4.08] | 2.87 – [0.8, 10.26] | 4.2 – [3.45, 5.11] |
| <2.0 g/dL | 0.85 – [0.62, 1.17] | 1.27 – [0.64, 2.51] | 1.91 – [0.65, 5.65] | 3.26 – [2.78, 3.82] |
| <2.1 g/dL | 0.74 – [0.56, 0.99] | 1.1 – [0.63, 1.91] | 1.67 – [0.68, 4.11] | 2.65 – [2.32, 3.03] |
| >2.1 g/dL | NA | NA | NA | NA |
|  |  |  |  |  |
| **Male** | **1-7 years** | **8-14 years** | **15-17 years** | **> 18 years** |
| <0.5 g/dL | 1.24 – [0.11, 13.71] | NA | NA | NA |
| <1.0 g/dL | 0.57 – [0.16, 2.0] | NA | NA | 42.2 – [4.71, 377.73] |
| <1.5 g/dL | 1.23 – [0.72, 2.09] | 2.8 – [0.31, 25.29] | NA | 12.52 – [6.55, 23.95] |
| <1.8 g/dL | 1.01 – [0.7, 1.46] | 2.48 – [1.0, 6.1] | 5.61 – [1.12, 28.07] | 4.2 – [3.32, 5.33] |
| <1.9 g/dL | 0.92 – [0.66, 1.27] | 2.02 – [1.01, 4.07] | 5.64 – [1.96, 16.18] | 3.4 – [2.82, 4.09] |
| <2.0 g/dL | 1.0 – [0.75, 1.33] | 1.73 – [0.97, 3.08] | 4.79 – [2.03, 11.31] | 2.71 – [2.33, 3.16] |
| <2.1 g/dL | 1.01 – [0.78, 1.3] | 1.3 – [0.8, 2.1] | 2.22 – [0.97, 5.09] | 2.1 – [1.85, 2.4] |
| >2.1 g/dL | NA | NA | NA | NA |

Confidence Interval = 95%; NA: Not Applicable
