# Supplementary material for: Calculated globulin as a surrogate marker for hypogammaglobulinemia: establishing clinical decision limits in a Brazilian population cohort
Source: Front Immunol. 2026 May 8;17:1743499. doi: 10.3389/fimmu.2026.1743499 (PMC13193802; doi:10.3389/fimmu.2026.1743499)
Supplement: Supplementary file 5 [file Table5.docx]

**Supplementary Table 5.** Prevalence of infections (%) - calculated globulin

| **Female** | - 1. **Years** | **8-14 years** | **15-17 years** | **> 18 years** |
| --- | --- | --- | --- | --- |
| 0-0.5 g/dL | 0 | 0 | NA | 0 |
| 0.5-1.0 g/dL | 0 | NA | NA | 0 |
| 1.0-1.5 g/dL | 8.10 | NA | 0 | 0 |
| 1.5-1.8 g/dL | 2.59 | 0 | 0 | 0.34 |
| 1.8-1.9 g/dL | 4.44 | 0 | 0 | 0 |
| 1.9-2.0 g/dL | 1.92 | 0 | 6.25 | 0 |
| 2.0-2.1 g/dL | 1.42 | 0 | 0 | 0.11 |
| >2.1 g/dL | 1.78 | 0.32 | 0.16 | 0.25 |
|  |  |  |  |  |
| **Male** | **1-7 years** | **8-14 years** | **15-17 years** | **> 18 years** |
| 0-0.5 g/dL | 0 | NA | NA | 0 |
| 0.5-1.0 g/dL | 0 | NA | NA | 0 |
| 1.0-1.5 g/dL | 6.25 | 0 | 0 | 6.25 |
| 1.5-1.8 g/dL | 3.52 | 0 | 0 | 1.24 |
| 1.8-1.9 g/dL | 0 | 0 | 0 | 0.31 |
| 1.9-2.0 g/dL | 10.93 | 2 | 0 | 0.37 |
| 2.0-2.1 g/dL | 3.77 | 0.88 | 0 | 0.46 |
| >2.1 g/dL | 1.99 | 0.39 | 0.19 | 0.27 |

NA: Not Applicable
