# Supplementary material for: Calculated globulin as a surrogate marker for hypogammaglobulinemia: establishing clinical decision limits in a Brazilian population cohort
Source: Front Immunol. 2026 May 8;17:1743499. doi: 10.3389/fimmu.2026.1743499 (PMC13193802; doi:10.3389/fimmu.2026.1743499)
Supplement: Supplementary file 6 [file Table6.docx]

**Supplementary Table 6.** Median use of antibiotics - calculated globulin

| **Female** | - 1. **Years** | **8-14 years** | **15-17 years** | **> 18 years** |
| --- | --- | --- | --- | --- |
| 0-0.5 g/dL | 2 | NA | 0 | 1 |
| 0.5-1.0 g/dL | 1.4* | 0 | 0 | 3* |
| 1.0-1.5 g/dL | 0.56* | 0 | 2 | 0.61* |
| 1.5-1.8 g/dL | 0.12 | NA | 0.08 | 0.15* |
| 1.8-1.9 g/dL | 0.25 | 0.29* | 1.4* | 0.12* |
| 1.9-2.0 g/dL | 0.03 | 0.06 | 0.06 | 0.07* |
| 2.0-2.1 g/dL | 0.24 | 0.06 | NA | 0.06 |
| >2.1 g/dL | 0.16 | 0.04 | 0.03 | 0.05 |
|  |  |  |  |  |
| **Male** | **1-7 years** | **8-14 years** | **15-17 years** | **> 18 years** |
| 0-0.5 g/dL | 1.66* | 0 | 0 | 0 |
| 0.5-1.0 g/dL | 0.92* | 0 | 0 | 2.75* |
| 1.0-1.5 g/dL | 0.72* | 1.2* | NA | 0.65* |
| 1.5-1.8 g/dL | 0.22* | 0.1* | NA | 0.26* |
| 1.8-1.9 g/dL | 0.07 | NA | NA | 0.21* |
| 1.9-2.0 g/dL | 0.26* | NA | 0.04 | 0.11 |
| 2.0-2.1 g/dL | 0.08 | NA | 0.12 | 0.11 |
| >2.1 g/dL | 0.16 | 0.03 | 0.04 | 0.08 |

Mann Whitney test, *p<0.05. NA: Not Applicable
