# Supplementary material for: Calculated globulin as a surrogate marker for hypogammaglobulinemia: establishing clinical decision limits in a Brazilian population cohort
Source: Front Immunol. 2026 May 8;17:1743499. doi: 10.3389/fimmu.2026.1743499 (PMC13193802; doi:10.3389/fimmu.2026.1743499)
Supplement: Supplementary file 7 [file Table7.docx]

**Supplementary Table 7**: Mean number of laboratory visits per patient according to age, sex, and calculated globulin (CG) levels. No significant differences were observed across groups.

| **Female** | - 1. **Years** | **8-14 years** | **15-17 years** | **> 18 years** |
| --- | --- | --- | --- | --- |
| 0-0.5 g/dL | 2 | 1 | 0 | 1 |
| 0.5-1.0 g/dL | 1 | 0 | 0 | 2.75 |
| 1.0-1.5 g/dL | 1.30 | 0 | 1 | 1.79 |
| 1.5-1.8 g/dL | 1.18 | 1.05 | 1.75 | 1.66* |
| 1.8-1.9 g/dL | 1.31 | 1.41 | 2.40 | 1.76 |
| 1.9-2.0 g/dL | 1.10 | 1.18 | 1.31 | 1.79* |
| 2.0-2.1 g/dL | 1.29 | 1.28 | 1.50 | 1.81* |
| >2.1 g/dL | 1.24 | 1.40 | 1.46 | 2.13 |
|  |  |  |  |  |
| **Male** | **1-7 years** | **8-14 years** | **15-17 years** | **> 18 years** |
| 0-0.5 g/dL | 1.67 | 0 | 0 | 1.00 |
| 0.5-1.0 g/dL | 1.23 | 0 | 0 | 1.75 |
| 1.0-1.5 g/dL | 1.62 | 1.80 | 1.00 | 1.47 |
| 1.5-1.8 g/dL | 1.42 | 1.21 | 1.50 | 1.50* |
| 1.8-1.9 g/dL | 1.27 | 1.38 | 1.27 | 1.58* |
| 1.9-2.0 g/dL | 1.12 | 1.14 | 1.09 | 1.52* |
| 2.0-2.1 g/dL | 1.23 | 1.27 | 1.44 | 1.65* |
| >2.1 g/dL | 1.21 | 1.42 | 1.46 | 1.96 |

Mann Whitney test, *p<0.05
