# Supplementary material for: Calculated globulin as a surrogate marker for hypogammaglobulinemia: establishing clinical decision limits in a Brazilian population cohort
Source: Front Immunol. 2026 May 8;17:1743499. doi: 10.3389/fimmu.2026.1743499 (PMC13193802; doi:10.3389/fimmu.2026.1743499)
Supplement: Supplementary file 8 [file Table8.docx]

**Supplementary Table 8.** Prevalence (%) of the use of human immunoglobulin according to sex, age group and levels of calculated globulin

| **Female** | - 1. **Years** | **8-14 years** | **15-17 years** | **> 18 years** |
| --- | --- | --- | --- | --- |
| 0-0.5 g/dL | 0 | 0 | NA | 0 |
| 0.5-1.0 g/dL | 40.00* | NA | NA | 25.00* |
| 1.0-1.5 g/dL | 8.10* | NA | 0 | 2.94* |
| 1.5-1.8 g/dL | 0 | 0 | 0 | 0.34 |
| 1.8-1.9 g/dL | 0 | 3.7 | 20.00* | 0.32 |
| 1.9-2.0 g/dL | 0 | 0 | 0 | 0 |
| 2.0-2.1 g/dL | 2.85 | 1.31 | 0 | 0.11 |
| >2.1 g/dL | 2.00 | 0.32 | 0.16 | 0.06 |
|  |  |  |  |  |
| **Male** | **1-7 years** | **8-14 years** | **15-17 years** | **> 18 years** |
| 0-0.5 g/dL | 0 | NA | NA | 0 |
| 0.5-1.0 g/dL | 7.69 | NA | NA | 0 |
| 1.0-1.5 g/dL | 4.16 | 0 | 0 | 3.12* |
| 1.5-1.8 g/dL | 1.17 | 0 | 0 | 1.55* |
| 1.8-1.9 g/dL | 0 | 0 | 0 | 0.94* |
| 1.9-2.0 g/dL | 1.56 | 0 | 0 | 0.37 |
| 2.0-2.1 g/dL | 0.94 | 0 | 2.56 | 0.34 |
| >2.1 g/dL | 1.45 | 0.39 | 0.38 | 0.14 |

Mann Whitney test, *p<0.05
